# Exacerbating and reversing lysosomal storage diseases: from yeast to humans

**DOI:** 10.15698/mic2017.09.588

**Published:** 2017-08-25

**Authors:** Tamayanthi Rajakumar, Andrew B. Munkacsi, Stephen L. Sturley

**Affiliations:** 1 School of Biological Sciences, Victoria University of Wellington, Wellington, New Zealand 6012.; 2 Centre for Biodiscovery, Victoria University of Wellington, Wellington, New Zealand 6012.; 3 Department of Genetics and Development, Columbia University Medical Center, New York, NY 10032.

**Keywords:** lysosomal storage disease, Niemann Pick Type-C disease, gene modifier, exacerbate-reverse, yeast, HIV, Ebola

## Abstract

Lysosomal storage diseases (LSDs) arise from monogenic deficiencies in lysosomal proteins and pathways and are characterized by a tissue-wide accumulation of a vast variety of macromolecules, normally specific to each genetic lesion. Strategies for treatment of LSDs commonly depend on reduction of the offending metabolite(s) by substrate depletion or enzyme replacement. However, at least 44 of the ~50 LSDs are currently recalcitrant to intervention. Murine models have provided significant insights into our understanding of many LSD mechanisms; however, these systems do not readily permit phenotypic screening of compound libraries, or the establishment of genetic or gene-environment interaction networks. Many of the genes causing LSDs are evolutionarily conserved, thus facilitating the application of models system to provide additional insight into LSDs. Here, we review the utility of yeast models of 3 LSDs: Batten disease, cystinosis, and Niemann-Pick type C disease. We will focus on the translation of research from yeast models into human patients suffering from these LSDs. We will also discuss the use of yeast models to investigate the penetrance of LSDs, such as Niemann-Pick type C disease, into more prevalent syndromes including viral infection and obesity.

## INTRODUCTION

Neurodegenerative diseases represent one of the leading causes of disability in modern populations. Approximately five million Americans currently have Alzheimer’s disease, a number that is expected to be as high as 13.4 million by 2050 1. It has recently become apparent that lysosomal function, through a variety of mechanisms, is a contributor to polygenically-determined neurodegenerative states and often causal in terms of certain monogenic cognitive disorders. Indeed, the vast majority of lysosomal storage disorders (LSDs) have neurological decay as a primary presentation or as a common sequel 2,3. The concept of LSDs, which are characterized by aberrant, excessive storage of cellular material in lysosomes, followed the discovery of a lysosomal enzyme deficiency (acid alpha-glucosidase) as the cause of Glycogen Storage Disease type II or Pompe disease in 1963. Great strides have since been made in understanding the biology of LSDs. Defective lysosomal storage typically occurs in many cell types, but the nervous system is particularly vulnerable to lysosomal mis-function, being affected in two-thirds of all LSDs. This review provides a summary of some of the better characterized LSDs and the pathways affected in these disorders.

Unfortunately, very few neurodegenerative conditions are treatable, and most worsen over time. Even monogenic neurodegenerative conditions, such as certain LSDs, where the biochemical and genetic etiology is very well defined, often remain tragically incurable, leaving palliative care as the only option to most patients. A novel and innovative rationale to treatment identification is clearly required. We propose that neuronal health is determined by very basic metabolic pathways, common to all cells and evolutionarily conserved from yeast to humans. Research models for polygenic disorders are hard to create and even harder to manipulate. By contrast, numerous spontaneous or induced models of rare monogenic disorders of high penetrance exist. These are frequently revealing with regard to disease mechanism and often establish therapeutic strategies for related polygenic pathologies (for example the LDL-Receptor and hypercholesterolemia). We have established a novel and innovative “exacerbate-reverse” approach to identify and exploit genetic loci that render cells sensitive to loss of genes defective in human monogenic diseases. We have successfully accomplished this strategy first in yeast and subsequently in mammalian cultured cell systems and murine models 4,5. Described here is the rationale behind this strategy, particularly with respect to the treatment of LSDs. Our major focus within this disease category is Niemann Pick type C (NP-C) disease, a classical LSD, typified by the accumulation of multiple lipids in the lysosomal compartment 6,7. We have established clear proof of principle in terms of extending our findings in yeast to cultured human patient fibroblasts and more recently to a murine model of NP-C disease 4,5. Ultimately, we propose to pursue these targets in human trials. Interestingly, NP-C disease, while itself a true orphan disorder, typifies the advantages of a studying rare diseases caused by unimolecular causation; variation in the NPC1 protein is associated with obesity risk and susceptibility to a variety of viral infections. In this fashion, research into an orphan disorder has the potential to reach beyond low prevalence and impact the health of millions.

## “EXACERBATE-REVERSE”: A model system based strategy for LSD intervention 

The availability of a sequenced budding yeast (*Saccharomyces cerevisiae*) genome for the past 20 years or more has rendered what is now a relatively common application of global genome-wide screens to comprehensively place gene products and their associations in the context of whole cell physiology and pathophysiology 8. The true appeal of a genetic screen is that it is generally accomplished without bias and thus highlights unanticipated avenues of intervention. Moreover, the conservation of sequence and function between yeast and human cells has elevated this model microbial eukaryote to a level unanticipated by many in terms of establishing human disease treatment strategies. We have chosen to focus solely on those interactions that are strongly conserved evolutionarily, biologically compelling, and amenable to pharmacological intervention. The latter parameter reflects our intent to achieve treatment of these diseases as rapidly as possible.

Numerous genetic strategies have been employed in yeast, probably more than any other model system, to identify and analyze interacting pathways. These initially took the shape of classical mutagenic approaches to suppressor and synthetic interaction screens, but were rapidly superceded by the availability of comprehensive, single deletion libraries of the whole yeast genome. For decades, sophisticated and systematic genome-wide experiments have been more feasible in yeast than in any other eukaryote 9,10. This is due largely to yeast having been the first eukaryotic genome sequenced in 1996, which resulted in the comprehensive identification of functional open reading frames (ORFs) and exceptionally elaborate gene annotation 11. As a result, genome-wide libraries of gene deletion strains, gene knockdown strains, overexpressed genes, and GFP-tagged proteins were constructed and used as community resources to investigate genetic and environmental perturbations. These deletion collections have been applied in numerous instances to understanding gene function, gene-environment and gene-gene (epistatic) interactions 12. These strategies identified orthologous genes, of which ~1000 genes are functionally conserved with human counterparts, variation in which is often associated with human disease 12,13.

**Figure 1:**
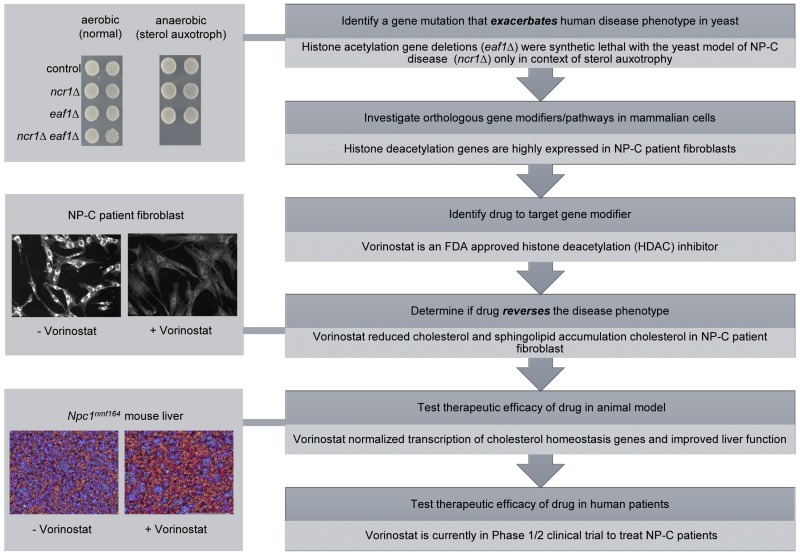
FIGURE 1: Exacerbate-reverse as an approach to identify genes and drugs to target and treat LSDs. The general outline of the approach (dark grey) starts with using genome-wide analyses in yeast to identify gene mutants that exacerbate the phenotype that mimics the human disease, followed by analyses of mammalian orthologs that can ultimately be targeted with drugs to reverse the disease phenotype. The original use of the approach investigating LSDs started with the yeast model of NP-C disease (light grey) and culminated in the identification of Vorinostat as a candidate therapy that is now in clinical trial. Dilutions of yeast cells were grown in aerobic (normal) and anaerobic (sterol auxotrophy) conditions. The NP-C patient fibroblasts were stained with filipin, a diagnostic marker of cholesterol accumulation. The *Npc1^nmf164 ^*mouse liver cells were stained with hematoxylin/eosin (H/E), to distinguish lipid-laden cells (blue) from healthy hepatocytes (red). Originally published in 14; © the American Society for Biochemistry and Molecular Biology.

An approach we have termed “*exacerbate-reverse”*, represents an extension of a classical “synthetic lethality” screen that we propose is applicable to any rare monogenic human disease for which there is a model system 4. We have perfected it for use in budding yeast (Figure 1), but any model organism that permits genetic screens of epistasis could be utilized. By this strategy, a yeast model of a human disease is screened against a genome-wide library of genetic variants (*e.g.*, a library of gene deletions, or overexpressed genes) and genetic modifiers are identified as a consequence of exacerbation of the disease associated phenotype. This epistatic screen for genetic interactions is rendered disease specific by performing the screen under conditions that reflect or are known to impact the human disease state. The synthetic interactions, most often observed as a conditional inviability of the mutant combinations, are then targeted in the opposite direction to genetically or pharmacologically reverse disease phenotypes in a mammalian model of the human disease. It is this reversal stage of the process that leads to therapeutic interventions that are made relevant to human disease by only targeting conserved and “druggable” pathways, ideally with FDA approved agents.

In contrast to “exacerbate-reverse”, a “rescue-reverse” approach can also be utilized. In this conceptually similar approach, gene deletions that directly rescue (*i.e.*, suppress) the disease severity of the LSD yeast model are identified. Accordingly, these reactions become potential drug targets whereby inhibitors would be predicted to reverse the disease phenotype in human cells. This strategy is limited to those disease models in which deletion of the ortholog has a clear phenotype in yeast.

## LYSOSOMES AND VACUOLES

Lysosomes are characteristic of all eukaryotic cells 15,16. This sac-like organelle is filled with numerous hydrolytic enzymes, and surrounded with lipoprotein membranes 15,17,18. There are approximately 120 lysosomal membrane proteins and 60 proteins present in the lysosomal matrix. The majority of the latter are acid hydrolases that require an acidic environment to degrade specific substrates 18,19. Lysosomes thus play a crucial role in the concerted action of selective and non-selective degradation of cargo 20,21.

Lysosomes are central to the interacting processes of recycling and degradation, which ultimately regulate other cellular processes and impact the overall homeostasis of the cell. In all cases, the by-products of lysosomal degradation are utilized by the cell after being trafficked out of the digestive compartment 22. Dysfunction of lysosomes initiates critical signalling cascades that can induce cell death mechanisms such as apoptosis and autophagy 21,23,24. Cell death in this case is largely a consequence of lysosomal accumulation of cargo due to defective degradation and recycling 21,25. Due to its role in various cellular processes, comprehending the function of lysosomes provides valuable insight into many diverse common and orphan disorders.

The vacuole, the fungal counterpart of the mammalian lysosome, represents a molecularly well-defined compartment maintained via distinctive ionic homeostasis 26. Approximately, 200 out of the 6000 genes in the yeast genome are annotated to impact vacuolar function based on either protein activity or localization of GFP-tagged protein markers 27. 27% of these 200 proteins are annotated as transporters with the remainder implicated in protein sorting and trafficking, membrane fusion, macromolecule degradation and organelle acidification 28,29. Minimally 45 genes have been implicated in cargo trafficking pathways from Golgi to vacuole in yeast via isolation of mutants with missorted soluble vacuolar proteins, deficient vacuolar protease activity or abnormal vacuolar morphology 30,31,32,33,34,35. Many such pathways and their mediators identified in these yeast vacuolar screens have functional orthologues in higher eukaryotes 36. For example, five distinctive classes of endosomal sorting complex required for transport (ESCRT) and the role of mono-ubiquitination as a sorting signal were first elucidated via exquisite studies on the yeast vacuole 37,38,39. This conservation allows researchers to confidently study the molecular basis of human disease, translating findings from yeast models to mammalian cells.

## LYSOSOMAL STORAGE DISEASES

There are approximately 50 lysosomal storage diseases (LSDs) arising from deficiencies in non-enzymatic lysosomal proteins, soluble lysosomal proteins or non-lysosomal proteins 40. Defects in proteins in the lysosomal lumen or lysosomal membrane as well as non-lysosomal proteins affect lysosomal function and result in the accumulation of macromolecules that are normally degraded in lysosomes 41,42. Overall, the prevalence of LSDs is rare at ~1:8000 births 43. The most frequently affected enzymes are sulfatases and glycosidases that degrade glycoconjugates such as gylcosylaminoproteoglycans, glycolipids and glycoproteins 44,45. In addition to a specific hydrolytic deficiency, interrupted vesicular trafficking and protein sorting also cause LSDs 40. Typically, LSDs are inherited as monogenic autosomal recessive traits and the symptoms of the disorder depend on the degree of residual function of the defective protein 42,44. There is extensive diversity among the LSDs with symptoms that initiate *in utero* or infancy on a continuous spectrum to symptoms that do not become apparent until late childhood or adulthood 43,44. The accumulation of substrates in LSDs occurs mostly in visceral tissues and the central nervous system, thus LSDs often lead to physical and neurological defects that shorten lifespan 45. For a minority of the LSDs, significant progress has been made to develop therapies that have been ultimately approved by federal agencies. However, the medical needs for most LSDs remain unfulfilled.

The general approach to candidate therapies has been to identify and/or develop drugs that restore function of the defective protein or limit the accumulation of the toxic metabolite by substrate depletion 46,47,48. To do this, it is pertinent to understand the specific function of the protein. Naturally occurring animal models (rabbit, sheep, dogs, cats) as well as genetically engineered mouse models have been vital to investigating gene function and pathogenesis 49,50,51. Genetically engineered mouse models are more commonly investigated than naturally occurring animal models, likely a consequence of pragmatic issues such as regular access to relatively inexpensive small animal facilities, and malleable genotypic diversity resulting in modulation of gene expression both temporally and spatially 50. The majority of animal models generally exhibit similar biochemical, molecular and physiological pathology to human patients 51. Overall, mammalian models contribute largely in revealing the disease biology and are critical as a precursor to therapeutic trials. However, mouse models exhibit extensive variation in the presentation of disease severity depending on genetic background 52,53,54. LSD-causing genes are generally well conserved among eukaryotes, and thus the underlying molecular mechanisms associated with LSDs can be meaningfully studied using genetic model organisms. Less complex eukaryotes with complete sequenced genomes are actually ideal to study LSDs since the orthologous genes and pathways involved in the disease are amenable to advanced genetic technology. For instance, the worm *Caenorhabditis elegans* contains 58 genes orthologous to human genes associated with LSDs (reviewed by 55). Other model organisms exploited to investigate LSDs are the fruitfly *Drosophila melanogaster *and various fungi including the filamentous plant pathogen* Fusarium graminearum, *the fission yeast *Schizosaccharomyces pombe*, and the budding yeast* S. cerevisiae *10,54,55. The latter organism is the focus of our own studies and of this review.

## BUDDING YEAST AS A GENETIC MODEL OF LSDs

Yeast models of human diseases have provided significant insight into many of the molecular mechanisms underlying cancer, neurodegenerative diseases, diabetes, and ageing 4,56,57,58,59,60,61,62. LSDs are no exception, given that 23 LSD-causing genes have orthologs in yeast based on amino acid sequence similarity (Table 1). The power of yeast as a model organism is not limited to conservation at the gene level but also extends to the pathway level; yeast models have identified candidate therapeutic pathways defined by genes that were not recognised as sequence orthologs 4. In this review, we will focus on LSDs currently recalcitrant to therapy, that arise from defects in lysosomal membrane proteins with functional yeast counterparts. We will describe the utilization of yeast as a genetic model to identify disease modifiers of three LSDs, cystinosis, Batten disease and Niemann Pick Type-C disease, and the manner in which this potentially leads to therapeutic interventions 4,63,64.

**Table 1:** **Yeast (*S. cerevisiae*) orthologs of human genes that encode lysosomal proteins associated with LSDs.** In total there are 23 yeast orthologs with <20% amino acid identity as defined using Gene2Function 13. *, human genes involved in viral infection.

**Human Gene**	**Human Disease**	**Function**	**Yeast Gene**	**% Identity**
AP3B1*	Hermansky-Pudlak Syndrome Type 2	Protein transport to late-Golgi/trans-Golgi network (TGN) and/or endosome	*APL6*	28
ATP13A2	Kufor-Rakep syndrome, Parkinson disease	Intracellular cation homeostasis	*YPK9*	35
ATP7A	Menke Disease	Copper transport	*CCC2*	33
CLN3	Neuronal ceroid lipofuscinosis, Batten Disease	Lysosomal pH homeostasis	*BTN1*	37
CLN8	Neuronal ceroid lipofuscinosis 8	Cell proliferation	*YPR114W*	24
CTNS	Cystinosis	Cystine/H^+^ transport	*ERS1*	29
CTSA	Galactosialidosis	Protein degradation	*PRC1*	30
CTSD	Neuronal ceroid lipofuscinosis 10	Protein degradation	*PEP4*	41
DHCR7*	Smith-Lemli-Optiz syndrome	Sterol biosynthesis	*ERG24*	32
DNAJC5	Neuronal ceroid lipofuscinosis 4 (CLN4) - adult CLN (Parry disease)	Exocytosis chaperone	*ERJ5*	33
GAA	Glycogen storage disease II (Pompe Disease)	Glycogen degradation	*ROT2*	27
GCH1	Dopa responsive dystonia	Nitric oxide synthesis	*FOL2*	58
GNPTG	Mucolipidosis III gamma	Carbohydrate phosporylation	*GTB1*	29
HRAS*	Costello Syndrome	Ras protein signal transduction	*RAS1* *RAS2*	61 61
LIPA	Wolman disease; infantile-onset autophagic vacuolar myopathy	Protein lipoylation	*YEH1* *YEH2*	27 25
MLL2	Kabuki Syndrome	Histone methyltransferase	*SET1*	35
NPC1*	Niemann-Pick type C	Cholesterol/sphingolipid transport	*NCR1*	33
NPC2*	Niemann-Pick type C	Cholesterol/sphingolipid transport	*NPC2*	24
POMT1	Walker-warburg syndrome	Glycosylation	*PMT4*	31
POMT2	Walker-warburg syndrome	Glycosylation	*PMT2*	34
PPT1	Neuronal Ceroid Lipofuscinosis 1	Cysteine modification	*CAX4*	23
SLC17A5	Sialuria; infantile sialic acid storage disorder	Sialic acid transport	*FEN2*	20
SMPD1*	Niemann-Pick type A	Sphingolipid metabolism	*PPN1*	24

### Cystinosis

**Figure 2:**
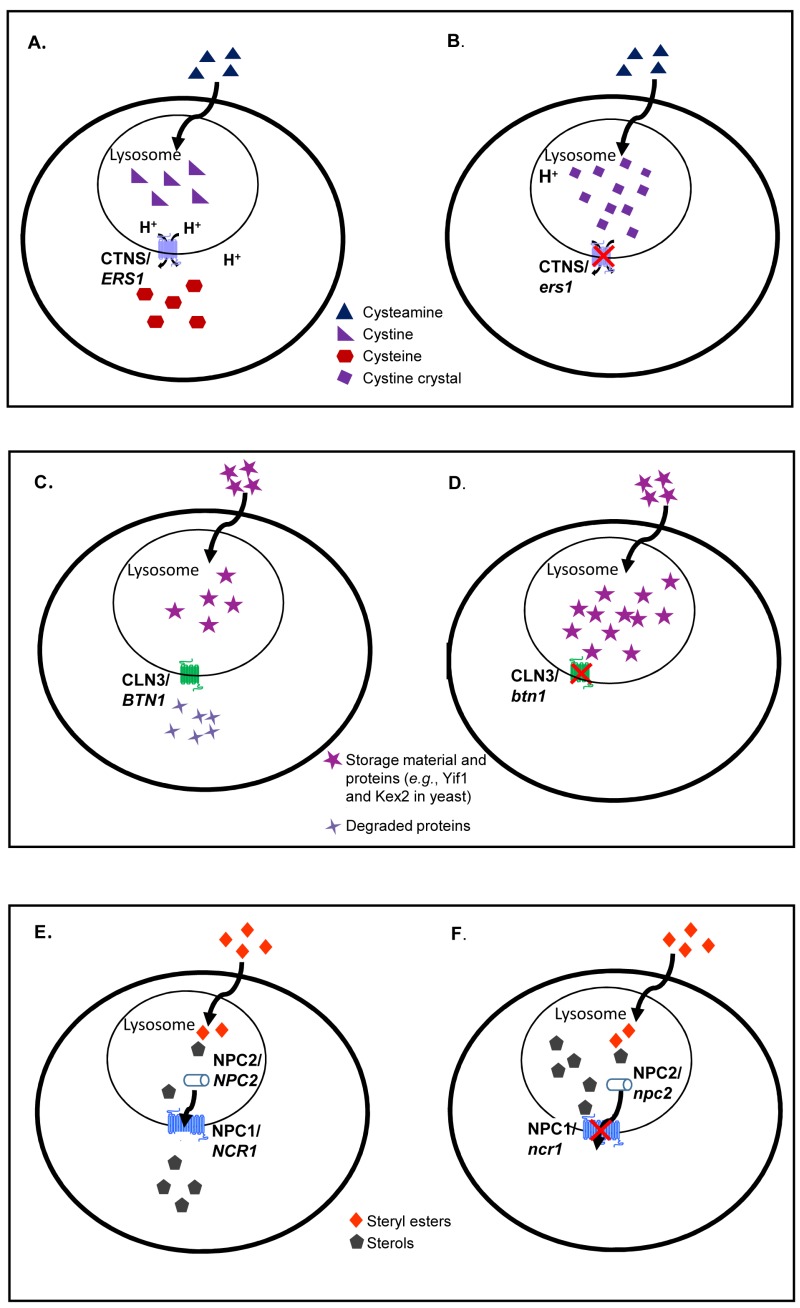
FIGURE 2: Comparisons of normal and mutant cells for three LSDs caused by lysosomal membrane protein deficiencies. In normal cells **(A)**, cystine is exported out of lysosomes to form cysteine. In cystinosis **(B)**, mutations in the disease-causing gene (CTNS, an ortholog of yeast *ERS1*) prevent the efflux of cystine out of lysosomes causing the formation of cystine crystal in the absence of functional cystinosin. In normal cells **(C)**, autofluorescent storage material and proteins are degraded and transported out of lysosomes. In Batten disease **(D)**, mutations in the disease-causing gene (CLN3, an ortholog of yeast *BTN1*) limit protein degradation causing an accumulation in the lysosome. In normal cells **(E)**, steryl esters are internalized by receptor mediated endocytosis, undergo lipolysis in the lysosome and egress from lysosomes as free sterols. In NP-C disease **(F)**, mutations in NPC1 (an ortholog of yeast *NCR1*) prevent the egress of sterol from the lysosome. The same disease phenotype is seen with NPC2 (the human ortholog of yeast *NPC2)* mutations. In an astounding example of evolutionary conservation, the yeast and human orthologs appear to be interchangeable for all three diseases.

Cystinosis is an autosomal recessive lysosomal storage disorder with the clinical hallmark of insoluble cysteine accumulation in the lysosome 65,66,67. Renal Fanconi syndrome, the most common inherited form of cystinosis in children 65,68,69, is caused by mutations in the *CTNS *gene, which encodes for cystinosin, a proton symporter that transports cystine out of the lysosomal compartment with proton-electromotive force generated by vacuolar (H+)-ATPase 64,70,71. Cystine, the disulphide linked form of cysteine, is thereby transported to the cytoplasm to be reduced into cysteine 72. In the disease state, due to a defect in efflux, insoluble cystine forms crystals that accumulate to toxic levels resulting in multiple organ dysfunction (Figure 2, A-B) 67. Depending on the severity of the *CTNS *gene mutation, the disorder has three different clinical presentations: infantile nephropathic, juvenile nephropathic and ocular non-nephropathic 65,71. The infantile nephropathic form presents as early as 6-8 months old and causes fatality by the age of 10 years old due to kidney failure 71. For the past 20 years, aminothiol cysteamine (β-mercaptoethylamine) has been used as treatment for this disorder as it diminishes the intralysosomal cystine via reduction of cystine and forms cysteine-mixed disulphide, which is then trafficked out of lysosomes through cationic amino acid transport 73,74. Overall, aminothiol cysteamine delays progression and prevents renal complications 75,76,77. However, the stringent dosing procedure and side effects of cysteamine therapy impose burdens on the patients. In addition, the cysteamine therapy requires an early diagnosis to ameliorate disease progression 78. Therefore, the search for a more effective treatment of this disorder remains active.

Whole animal and cultured mammalian cell models have been developed and utilized to further understand the pathogenic mechanisms in cystinosis 64,79,80. For example, disrupted vesicle trafficking and mTOR signalling were identified as pathophysiological events associated with human cystinosis but are unlikely to be causative 81,82. Despite extensive investigations of higher eukaryotes, the primary molecular mechanism of cystinosis remains vague. The human *CTNS *gene product is highly conserved with the Ers1p protein of *S. cerevisiae *(29% identical and 48% similar) 83 (Table 1). Moreover, yeast *ERS1* is a functional ortholog based on complementation of hygromycin B (hygB) sensitivity of *ers1Δ* deletion mutant strains by heterologous expression of human CTNS 84. Similar to cystinosin, yeast Ers1p localizes to endosomes and vacuoles 84. However, in contrast to *CTNS *mutants in mammalian cells, loss of *ERS1 *does not affect yeast viability and no accumulation of cystine is observed 84.

Given this conservation, the yeast model has provided significant insight into the molecular mechanisms underlying cystinosis particularly with respect to pathways that may modify cystine accumulation in patients. A comparative transcriptomic analysis of *ers1Δ *strains implicated sulfur homeostasis as aberrant, which was consistent with the gene expression profile of cystinotic cells from cystinosis patients. Additionally, energy production, oxidative stress, calcium and potassium transport and stress response were also awry in this mutant model 85. A high copy suppressor screen in yeast identified that *MEH1*, a gene critical for the process of microautophagy and the function of amino acid permease Gap1p that mediates cysteine uptake, suppressed hygB sensitivity in the *ers1Δ *yeast model 84,86. *MEH1* also was identified in an interaction with *GTR1*, a small Ras-related GTPase involved in macroautophagy 84. Further elucidating the role of *MEH1 *and* GTR1 *in yeast, as well as the human orthologs in mammalian cells, may provide more insight into the molecular mechanism of cystinosis. It would also be informative to identify gene deletions that suppress hygB sensitivity of *ers1Δ*, an experiment that can be easily conducted in yeast via a Synthetic Genetic Array (SGA) analysis wherein the *ers1Δ *strain is crossed with the deletion library to generate haploid double deletion strains. As hygB sensitivity in yeast is a marker for disease severity in humans, this analysis would provide a list of candidate genes and pathways that modify disease severity. More broadly, investigations of these modifiers may identify the compensatory mechanism(s) that prevent the accumulation of cystine in yeast and thus create a more powerful yeast model of cystinosis with a similar phenotype to humans.

### Batten Disease

Batten disease encompasses a group of fatal, pediatric, autosomal recessive lysosomal storage disorders that are the most common form of neuronal ceroid lipofuscinosis (NCL) 87,88,89. The age of onset can vary from congenital, infantile, late infantile, juvenile, to adult onset 90. Autofluorescent storage material, which contains high levels of subunit c of mitochondrial ATP synthase, accumulates in the lysosome of affected patients (Figure 2, C-D), although it is unclear whether this is causative or merely correlative 91. Mutations in any one of 14 genes confer NCL, with variation at the *CLN3 *locus being the most common cause of juvenile NCL, also known as Batten disease. 63,92,93. The *CLN3 *gene encodes a multipass transmembrane protein that localizes to the lysosomal, endosomal and autophagosomal compartments 94,95. The major role of Cln3p has not been completely elucidated, however it has been proposed to play a key role in vesicular trafficking pathways 96,97.

The obscure etiology of Batten disease warrants a new approach both to mechanism and treatment. Five of the 14 NCL genes (*CLN3, CLN8, CTSD, DNAJC5, *and *PPT1)* are conserved in yeast (Table 1). Although large animal (*e.g.*, sheep) and murine models have been developed to study the mechanism of juvenile NCL, the evolutionary conservation of *CLN3, *the most common cause of Batten disease*, *allows the use of lower eukaryotic genetic models to study the disorder 98,99. The Btn1p protein is the *S. cerevisiae* ortholog of human CLN3 sharing 37% amino acid identity 100. *BTN1* deletion strains exhibited increased plasma membrane ATPase activity, acidified growth media and resistance to D-(-)-threo-2-amino-1-[p-nitrophenyl]-1, 3-propanediol (ANP) 101. *BTN1 *is apparently not involved in degradation of mitochondrial ATPase subunit *c, *however expression of human CLN3, complements the ANP resistance of *btn1Δ *yeast, indicating that *BTN1* and CLN3 are functionally conserved 101,102. Interestingly, the degree of ANP resistance reflected the severity of the disorder imposed by expression of different mutant human alleles 100,101. Vacuolar and cytoplasmic arginine levels as well as ATP-dependent arginine uptake were further observed to be deficient in *btn1Δ *strains 103. Notably, lysosomes of human fibroblasts derived from JNCL patients also exhibited an elevated pH, suggesting a conserved interaction between pH homeostasis and arginine metabolism 103,104. An additional investigation of *btn1Δ *yeast strains revealed increased resistance to menadione-generated oxidative stress via impaired nitric oxide synthesis and decreased levels of arginine 105. The defects in arginine homeostasis that were identified in yeast may be an underlying mechanism of JNCL in humans.

Genome-wide screens have also been applied to advance our understanding of Batten disease using the *btn1Δ *yeast model. A Synthetic Genetic Array was conducted to systematically quantify the effect of ~4,800 independent gene deletions on the *btn1Δ *strain with little success initially; gene deletions that conferred lethality to the *btn1Δ *strain under basal conditions were not identified 106. However, under conditional screening conditions (altered media amino acid levels or pH), 18 genetic interactions were identified that implicated phospholipid distribution and endosome-Golgi retrograde transport 96,102,107,108,109,110 in Batten disease. These genetic interactions suggest that regulation of vacuolar cationic homeostasis could be disrupted in the *btn1Δ* yeast model, and may underlie the accumulation of storage material in Batten disease in humans. An alternative strategy would be to exploit the resistance of *btn1Δ *to ANP and screen *btn1Δ geneXΔ *double deletion mutants to identify gene deletions that reduce the resistance of *btn1Δ *to ANP. Likewise, gene overexpression screens to identify upregulated genes that reduce or increase the resistance of *btn1Δ *to ANP would identify putative therapeutic targets. Cumulatively, these genes would be modifiers of phenotypic severity in yeast, and orthologous genes and pathways would then be investigated as candidate disease modifiers in human cells.

### Niemann-Pick Type C Disease

Niemann-Pick type C (NP-C) disease (Figure 2, E-F), is a complex lipid lysosomal storage disorder, typified by accumulation of low-density lipoprotein-derived unesterified cholesterol, sphingomyelin, glucosylceramide and sphingosine 111. NP-C disease is inherited in a monogenic, autosomal recessive fashion due to independent defects in either of two genes, NPC1 or NPC2 6,7. Approximately 95% of cases are due to mutations in NPC1 and 5% are caused by mutations in NPC2 112. NPC1 is a ~13-pass transmembrane domain protein positioned in the limiting membrane of the lysosome while NPC2 is a soluble lysosomal protein 111. There are approximately 250 and 20 different disease-causing mutations in NPC1 and NPC2, respectively, that contribute to a wide range of variation in disease progression 113,114,115,116.

The NP-C proteins have been proposed as cholesterol transporters, in part because they both possess lipid-binding domains. However, the affinity of cholesterol for NPC1 is orders of magnitude lower than for NPC2, thus cholesterol binding to NPC1 may act catalytically, as opposed to bulk transfer or solubilisation 117,118. NP-C disease is further typified by the accumulation of sphingolipids 119. We previously hypothesized that NPC1 may be a rheostat that initiates transport of multiple lipids by other proteins and interacts with cellular signaling pathways to optimize membrane fluidity 7,120,121. The identification of these pathways, for example using a genetic-based screen, is key to understanding this syndrome and may reveal unanticipated therapeutic possibilities. Previously, we established budding yeast (*S. cerevisiae*) as a genetically tractable and valid model of NP-C disease by several criteria 122. Most importantly; rescue of the cholesterol and ganglioside trafficking defect in mammalian NPC1-deficient cell culture models was achieved by expression of the yeast NPC1 ortholog encoded by *NCR1* 4,8. Human NPC1 and yeast *NCR1* encode proteins with 33% sequence identity. This identity persists to specific homology domains such as the sterol sensing domain (SSD) and the *Patched* homology domains. In general, residues key to the activity of this protein, as defined by the occurrence of specific disease mutations in NP-C patients are also conserved. Moreover both yeast and mammalian NPC1 proteins localize to limiting membranes of the lysosomal (vacuolar)/endosomal system 122. Similarly, the yeast NPC2 protein is structurally and functionally orthologous to human NPC2 with 23% primary sequence identity and the ability to restore normal lipid homeostasis to human NPC2 patient fibroblasts. Interestingly, the yeast models of NP-C disease are distinct from other models of NP-C disease and have thus been exploited to capitalize on these distinctions 9,10,122. Similar to the *btn1Δ *and *ers1Δ *yeast LSD models, yeast strains deficient in *NCR1* do not exhibit a striking growth defect, although Vilaca and colleagues have implicated the *NCR1* mediated pathway in chronological lifespan (CLS), a measurement of non-replicative aging in yeast. Reduced CLS can be rescued with deletion of genes mediating sphingolipid metabolism further supporting a role of NCR1 in sphingolipid homeostasis 123. *NCR1*-deficient yeast cells do not display any obvious defect in sterol metabolism, although membrane microdomains and microautophagy are aberrant during quiescence 4,9,122,124,125,126. A sphingolipid related metabolic phenotype caused by a dominant negative mutation in *NCR1* prompted the hypothesis that the ancestral function of NPC1 was in recycling sphingolipids 122, and this was later supported with the observation of the accumulation of long chain sphingoid bases in an *ncr1Δ *yeast variant 123*. *The lack of a compelling null phenotype in yeast *NCR1* deletion strains, combined with our findings on conservation of these pathways between yeast and mammals provoked a series of screens for modifiers of *NCR1* in yeast 4. We performed numerous independent screens that assess pathway interactions representing approximately 6 fold-coverage of the yeast genome or the equivalent of 33,437 interactions. The most lucrative strategy required us to adapt our screens for genetic interactions to be conditional on the environment, based on the removal of oxygen to confer sterol auxotrophy. These conditions were deliberately chosen to mimic the context of NP-C disease with respect to defective sterol transport and homeostasis. This conditional, genome-wide Synthetic Genetic Array (SGA) analysis identified 13 gene deletions that reduced lifespan in the *ncr1Δ* strain only in the context of sterol auxotrophy (induced by anaerobiosis) 4. This strategy identified gene deletions in two histone acetylase genes that exacerbated inviability of *ncr1Δ* during anaerobic growth. This observation prompted the hypothesis that histone acetylase activity was reduced (and by inference histone deacetylase (HDAC) activity was increased) in human NP-C disease 4. Indeed, subsequent experiments demonstrated that expression levels of eight of 11 HDAC genes were increased in NP-C patient fibroblasts. Consequently a key question arose; would pharmacologically elevating histone acetylation in NPC1 mutant human fibroblasts alleviate any consequences of NPC1-deficiency? Strikingly, we found that inhibiting HDACs with suberoylanilide hydroxamic acid (SAHA, Vorinostat) dramatically reversed cholesterol and sphingolipid accumulation by 80% and 30%, respectively, and restored defective cholesterol esterification by 500% 4. This treatment completely eliminated the cellular basis of NP-C disease, a result that was independently confirmed by others 5,127,128,129. The recovery mechanism is not fully understood, but may partially include a chaperone mechanism that results in increased expression of NPC1 and reduced endoplasmic reticulum associated degradation pathways 129.

Our exacerbate-reverse approach identified Vorinostat as a candidate therapy in 2011 and Vorinostat was approved to treat cutaneous T-cell lymphoma in 2006. Hence a Vorinostat clinical trial was fast-tracked without proven therapeutic efficacy in an animal model of NP-C disease since there was no approved therapy to treat this fatal, pediatric disease. We subsequently tested two distinct murine models of NPC1 deficiency for their phenotypic response to HDAC inhibitors: The *Npc1^nih^* variant, defined by a null allele of Npc1, and *Npc1^nmf164^* (a missense allele of NPC1 comprising a *D1005G* protein variation); both animal lines develop a chronic, debilitating neurological disease and die between 9-12 and 13-16 weeks of age, respectively (the missense allele confers a milder, later onset, form of the disease). Using *Npc1^nmf164^* mice, we conducted a study to assess the efficiency of Vorinostat as potential therapeutic for NP-C disease. We determined that Vorinostat normalized transcriptional regulation of eight key genes critical for cholesterol homeostasis in the liver, thereby improving liver function and pathology 5. Importantly, Vorinostat treatment regulated metabolism of apolipoprotein B and the gene expression of key components of lipid homeostasis in primary hepatocytes from both *Npc1^nih^* and *Npc1^nmf164^* mice. This *in vivo* result is consistent with Vorinostat-mediated reduction in filipin fluorescence in 52 of 60 different NPC1 mutations in cultured cells 5, suggesting that Vorinostat can be used to treat symptoms of most NP-C patients particularly if blood-brain barrier penetration of Vorinostat can be improved 5.

## LYSOSOMAL FUNCTION AND PREVALENT DISEASES

As mentioned previously, aberrant lysosomal function and cellular degradation has implications for all cells and thus contributes significantly to both health and disease 25. Variation in the NPC1 protein clearly exemplifies this phenomenon with genetic penetrance beyond the orphan disorder of NP-C disease into several infectious diseases, atherosclerosis, obesity, and common neurodegenerative diseases. In what may be a prime example of heterozygote advantage, carrier status (for example in parents of affected NP-C children) can be protective from atherosclerosis and certain viral infections. Conversely, heterozygosity at the NP-C loci may be a disadvantage in disease states such as obesity and Alzheimer’s disease. The association of NP-C disease with atherosclerosis and tauopathies such as Alzheimer’s disease has previously been described 14,130. Indeed the similarities of NP-C disease to tauopathies are striking; purkinje cell death is accompanied by accumulation of paired helical filaments, neurofibrillary tangles, hyper-phosphorylated *tau* and amyloid-β peptides. Here we briefly review the importance of investigating NP-C disease to further understand AIDS, infectious haemorrhagic fever, and obesity. In particular we will describe the application of the aforementioned genetic strategies to establish novel approaches to therapeutic interventions for these high impact diseases.

Among the conserved LSD-causing genes in yeast, six genes were also associated with viral assembly, entry, and replication (Table 1) 131,132,133,134,135,136. Of particular note the NPC1 pathway, mainly because of its critical role in cholesterol homeostasis, facilitates a crucial stage in the infectious cycle of retroviruses such as human immunodeficiency virus (HIV), filoviruses such as Ebola **,** togaviruses such as Chikungunya, and Flaviviruses (including Zika, West Nile and Dengue virus) 131,132. To develop effective antiviral treatments, it is critical to fully understand virus-host interaction at the molecular level. Thus, the rare occurrence of NP-C disease and LSDs in general are suddenly relevant to the biology of “more common” diseases and integral to the development of treatments of HIV and Ebola.

### The role of the NPC1 protein in the life cycle of filoviruses

**Figure 3:**
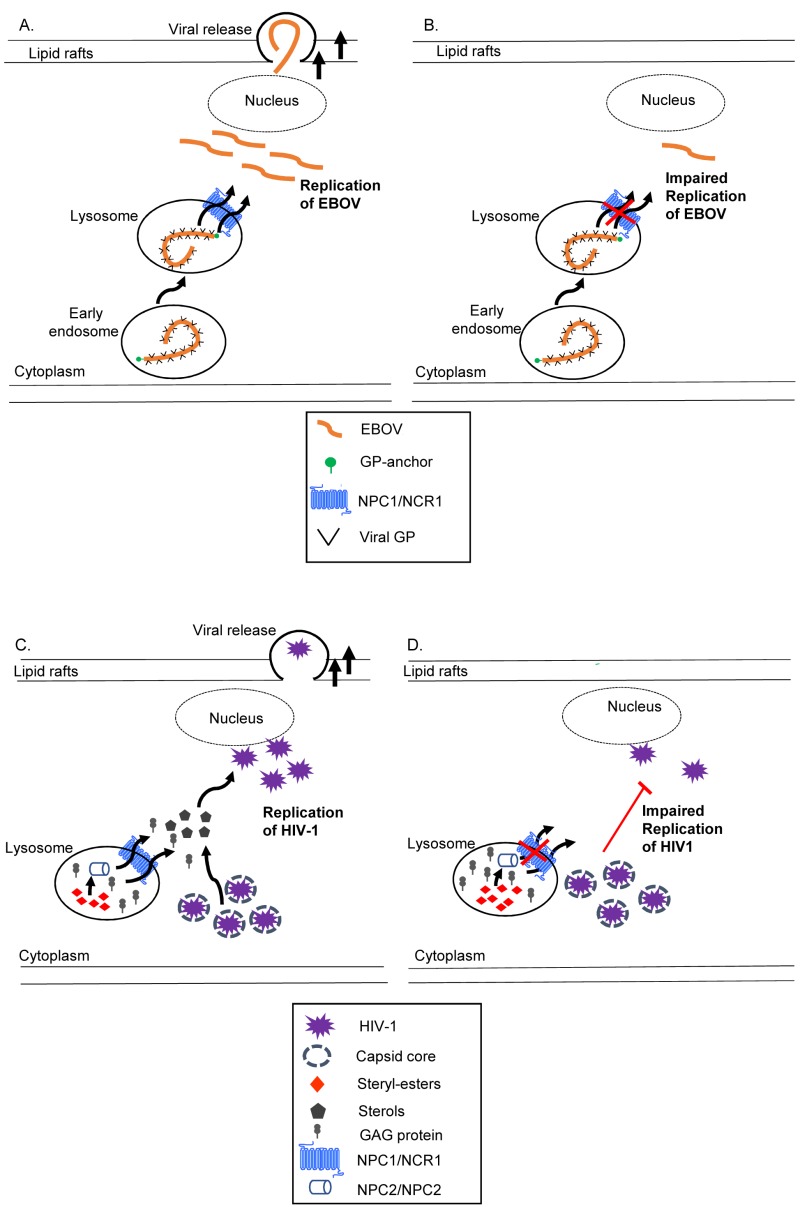
FIGURE 3: The current models for the role of NPC1 in replication of infectious viruses such as EBOV and HIV-1. *Infectious haemorrhagic fever* **(A-B)**. EBOV is transported to lysosomes at which point the virally encoded glycoprotein (GP) interacts with NPC1 to release nucleocapsid for replication and cytoplasmic release via lipid rafts 138. In NPC1 deficient states, the cytoplasmic release of EBOV is disrupted without efficient replication of EBOV. *Acquired Immunodeficiency Syndrome* **(C-D).** Free cholesterol egressed from the lysosome by NPC1 or NPC2 is a subset of the cholesterol pool utilized by HIV-1 for replication 143. Mutations in NPC1 reduce cholesterol availability and increase GAG protein accumulation, thereby impeding the cytoplasmic release and replication of HIV-1 virions.

Ebola viruses (EBOV) cause infectious haemorrhagic fever and are classified in the family of *Filoviridae *133. Filoviruses are enveloped, non-segmented, negative stranded RNA viruses with filamentous particles that require a virally encoded glycoprotein (GP) for the lysosomal exit and replication of the virus 134,135,136,137. In common with many viruses, cholesterol-sphingomyelin enriched microdomains (“lipid rafts”) are utilized by filoviruses to bud from cells and the status of these platforms can be compromised by NP-C pathway defects 137. Recent studies have found NPC1 specifically to be a crucial host factor required for EBOV infection 138,139. Interestingly, the direct involvement of NPC1 in EBOV infection is independent of both the egress of cholesterol and the NPC2 protein (Figure 3, A-B). Structural studies have elucidated the binding between NPC1 and viral GP, and provided a means to develop EBOV anti-infective agents that disrupt this physical interaction 140,141. Additional insight into the molecular mechanism of the virus GP-NPC1 interaction and infection pathogenesis is required to fully understand the role of NPC1 in EBOV infection. Since NPC1 is conserved as Ncr1p in yeast, a yeast model of negative-stranded RNA EBOV would comprise expression of the EBOV-viral RNA polymerase complex or EBOV RNA genome such that viral replication could occur 142. The components that are crucial for EBOV replication, their interacting host genes, and pathways mediating entry and replication of EBOV would then be interrogated with the power of various yeast mutant libraries in a comparison of wild-type yeast with the *ncr1Δ* yeast model of NP-C disease.

### The NP-C proteins and the life cycle of retroviruses

The NP-C pathway is also critical to the replication and infectivity of HIV-1, the causative agent for acquired immune deficiency syndrome (AIDS) in humans. HIV-1 is a complex retrovirus that is dependent on cholesterol as a cellular platform for replication 144,145,146. In common with filoviruses, the cellular entry, association, and budding of HIV-1 requires an intact NP-C pathway and membrane microdomains (rafts) enriched with cholesterol and sphingolipids 147,148,149. Accordingly, a decrease in cellular cholesterol depletes production of HIV-1 particles 150. Indeed, HIV-1 virus infectivity was affected by cholesterol efflux from the lysosome mediated by the NPC1/NPC2 pathway 151. The accessory protein of HIV-1, negative effector (Nef) activates multiple genes involved in cholesterol synthesis and thus increases the trafficking of cholesterol to viral particles and lipid rafts 143. Interestingly, Tang and co-workers observed impaired virus release as the sub-cellular cholesterol concentration increased in NPC1-mutant cells. This was coincident with accumulation of the HIV-1 Gag (group-specific antigen) protein required for structural integrity, maturation and replication of the retrovirus 152 (Figure 3. C-D). Despite these advances, the molecular role of the NP-C proteins in HIV-1 infection is not fully understood. Utilizing the power of yeast genetics and the *ncr1Δ*yeast model of NP-C disease is relatively straightforward given that different proteins encoding for HIV-1 (the reverse transcriptase, viral protein R, integrase, protease, and HIV-1 translation machinery) have been studied using yeast models (see review 153). Genome-wide analyses such as the sterol auxotrophy screen conducted for the *ncr1Δ* yeast model of NP-C disease would likely identify genes regulated by the HIV-1 genome as well as define genetic interactions and compensatory mechanisms that could reveal novel targets for anti-viral treatment.

### Interaction of NP-C Pathways with obesity

We hypothesize a pivotal role of the NP-C pathway, particularly the NPC1 protein, as a nexus in lysosomal lipid metabolism that prevents the lipid toxicity that underlies many metabolic disturbances such as obesity and diabetes. A convincing genome wide association study (GWAS; multiple populations, multiple polymorphisms, both genders; 11,12,13,15,16,17,18,19) demonstrated a link between variation at the NPC1 locus and risk for obesity and diabetes. In European populations, a GWAS identified three new alleles of NPC1 strongly associated with obesity 154. Similarly variation at the NPC2 locus has been associated with obesity in Korean populations 155. These observations have subsequently been recapitulated in several animal models of NP-C disease 156,157. The mechanism behind this robust genetic association of NP-C pathway deficiency with obesity is currently unknown and could be further investigated by the integration of yeast models of obesity and NP-C disease 158.

## CONCLUSION AND PERSPECTIVES BEYOND LSDs

In summary, the rapid and continuing advancement of genomic and proteomic research in yeast is a key advantage to identifying genetic modifiers as therapeutic drug targets. *S. cerevisae *is a simple eukaryotic model that generates biochemical and genetic output that can readily be translated into more complex eukaryotes. Hence, screening for monogenically determined disease modifiers using a yeast LSD model and translating it into higher eukaryotes saves time and cost in identifying the therapeutic drug target. Although there are some limitations encountered when using a yeast model of LSDs, we demonstrated these can be overcome by using experimental conditions that mimic the processes defective in human patients. The “exacerbate-reverse” approach described here has great potential to contribute to our understanding of LSDs with specific emphasis on drug discovery. Our discovery of Vorinostat as a candidate therapy now in clinical trial has established this strategy as a promising pipeline to LSD treatments 4. Similarly, this approach can be utilized with the *btn1Δ* yeast model of Batten disease and ANP resistance to identify modifiers of Batten disease and to the *ers1Δ* yeast model of cystinosis via a “rescue-reverse” of hygB sensitivity as potential drug targets to treat cystinosis. Apart from discovery of drug targets to treat LSDs, yeast is a powerful tool to study virus infection and pathogenesis. The molecular mechanism of virus-host interactions and the characterization of the host functions can be easily elucidated using the genetic tools available. Remarkably, interaction of each individual viral-encoding protein can easily be studied in yeast via heterologous expression of each protein in yeast, which can then be integrated into powerful genome-wide analyses of various mutant libraries. These analyses would direct the discovery of antiviral treatments by identifying genes that prevent virus entry or replication. In summary, genome-wide analyses that exacerbate or ameliorate yeast growth phenotypes caused by yeast gene mutations or viral proteins may prove advantageous to identify genes and pathways that modify human genetic diseases and infection pathogenesis.

## References

[1] Centers for Disease Control and Prevention Healthy Brain Initiative: Alzheimer’s Disease, Healthy Aging.. https://www.cdc.gov/aging/aginginfo/alzheimers.htm.

[2] Onyenwoke RU, Brenman JE (2016). Lysosomal Storage Diseases - Regulating Neurodegeneration.. J Exp Neurosci.

[3] Boustany R-MN (2013). Lysosomal storage diseases - the horizon expands.. Nat Rev Neurol.

[4] Munkacsi AB, Chen FW, Brinkman MA, Higaki K, Gutiérrez GD, Chaudhari J, Layer JV, Tong A, Bard M, Boone C, Ioannou YA, Sturley SL (2011). An “exacerbate-reverse” strategy in yeast identifies histone deacetylase inhibition as a correction for cholesterol and sphingolipid transport defects in human Niemann-Pick type C disease.. J Biol Chem.

[5] Munkacsi AB, Hammond N, Schneider RT, Senanayake DS, Higaki K, Lagutin K, Bloor SJ, Ory DS, Maue RA, Chen FW, Hernandez-Ono A, Dahlson N, Repa JJ, Ginsberg HN, Ioannou YA, Sturley SL (2017). Normalization of hepatic homeostasis in the Npc1nmf164 mouse model of Niemann-Pick type C disease treated with the histone deacetylase inhibitor Vorinostat.. J Biol Chem.

[6] Sturley SL, Patterson MC, Balch W, Liscum L (2004). The pathophysiology and mechanisms of NP-C disease.. Biochim Biophys Acta.

[7] Munkacsi AB, Porto AF, Sturley SL (2007). Niemann-Pick type C disease proteins: orphan transporters or membrane rheostats?. Future Lipidol.

[8] Dobzhansky T (1946). Genetics of Natural Populations. Xiii. Recombination and Variability in Populations of Drosophila pseudoobscura.. Genetics.

[9] Somers KL, Wenger DA, Royals MA, Carstea ED, Connally HE, Kelly T, Kimball R, Thrall MA (1999). Complementation studies in human and feline Niemann-Pick type C disease.. Mol Genet Metab.

[10] Lopez ME, Scott MP (2013). Genetic dissection of a cell-autonomous neurodegenerative disorder: lessons learned from mouse models of Niemann-Pick disease type C.. Dis Model Mech.

[11] Hong EL, Balakrishnan R, Dong Q, Christie KR, Park J, Binkley G, Costanzo MC, Dwight SS, Engel SR, Fisk DG, Hirschman JE, Hitz BC, Krieger CJ, Livstone MS, Miyasato SR, Nash RS, Oughtred R, Skrzypek MS, Weng S, Wong ED, Zhu KK, Dolinski K, Botstein D, Cherry JM (2008). Gene Ontology annotations at SGD: new data sources and annotation methods.. Nucleic Acids Res.

[12] Heinicke S, Livstone MS, Lu C, Oughtred R, Kang F, Angiuoli SV, White O, Botstein D, Dolinski K (2007). The Princeton Protein Orthology Database (P-POD): A Comparative Genomics Analysis Tool for Biologists.. PLoS ONE.

[13] Hu Y, Comjean A, Mohr SE, FlyBase Consortium, Perrimon N (2017). Gene2Function: An Integrated Online Resource for Gene Function Discovery.. G3 (Bethesda).

[14] Zhang JR, Coleman T, Langmade SJ, Scherrer DE, Lane L, Lanier MH, Feng C, Sands MS, Schaffer JE, Semenkovich CF, Ory DS (2008). Niemann-Pick C1 protects against atherosclerosis in mice via regulation of macrophage intracellular cholesterol trafficking.. J Clin Invest.

[15] Armstrong J (2010). Yeast vacuoles: more than a model lysosome.. Trends Cell Biol.

[16] de Duve C, Pressman BC, Gianetto R, Wattiaux R, Appelmans F (1955). Tissue fractionation studies. 6. Intracellular distribution patterns of enzymes in rat-liver tissue.. Biochemical J.

[17] Hoffman-Sommer M, Rytka J (2007). The yeast protein sorting pathway as an experimental model for lysosomal trafficking.. Expert Rev Clin Immunol.

[18] Braulke T, Bonifacino JS (2009). Sorting of lysosomal proteins.. Biochim Biophys Acta.

[19] Kollmann K, Mutenda KE, Balleininger M, Eckermann E, von Figura K, Schmidt B, Lubke T (2005). Identification of novel lysosomal matrix proteins by proteome analysis.. Proteomics.

[20] Janvier K, Bonifacino JS (2005). Role of the Endocytic Machinery in the Sorting of Lysosome-associated Membrane Proteins.. Mol Biol Cell.

[21] Appelqvist H, Waster P, Kagedal K, Ollinger K (2013). The lysosome: from waste bag to potential therapeutic target.. J Mol Cell Biol.

[22] Lloyd JB (1996). Metabolite efflux and influx across the lysosome membrane.. Subcell Biochem.

[23] Li X, Rydzewski N, Hider A, Zhang X, Yang J, Wang W, Gao Q, Cheng X, Xu H (2016). A molecular mechanism to regulate lysosome motility for lysosome positioning and tubulation.. Nat Cell Biol.

[24] Turk B, Turk V (2009). Lysosomes as “suicide bags” in cell death: myth or reality?. J Biol Chem.

[25] Boya P (2012). Lysosomal function and dysfunction: mechanism and disease.. Antioxid Redox Signal.

[26] Li SC, Kane PM (2009). The yeast lysosome-like vacuole: Endpoint and crossroads.. Biochim Biophys Acta.

[27] Huh W-K, Falvo JV, Gerke LC, Carroll AS, Howson RW, Weissman JS, O’Shea EK (2003). Global analysis of protein localization in budding yeast.. Nature.

[28] Blott EJ, Griffiths GM (2002). Secretory lysosomes.. Nat Rev Mol Cell Biol.

[29] Hecht KA, O’Donnell AF, Brodsky JL (2014). The proteolytic landscape of the yeast vacuole.. Cell Logist.

[30] Banta LM, Robinson JS, Klionsky DJ, Emr SD (1988). Organelle assembly in yeast: characterization of yeast mutants defective in vacuolar biogenesis and protein sorting.. J Cell Biol.

[31] Robinson JS, Klionsky DJ, Banta LM, Emr SD (1988). Protein sorting in Saccharomyces cerevisiae: isolation of mutants defective in the delivery and processing of multiple vacuolar hydrolases.. Mol Cell Biol.

[32] Rothman JH, Stevens TH (1986). Protein sorting in yeast: mutants defective in vacuole biogenesis mislocalize vacuolar proteins into the late secretory pathway.. Cell.

[33] Rothman JH, Howald I, Stevens TH (1989). Characterization of genes required for protein sorting and vacuolar function in the yeast Saccharomyces cerevisiae.. EMBO J.

[34] Jones EW (1977). Proteinase mutants of Saccharomyces cerevisiae.. Genetics.

[35] Raymond CK, Howald-Stevenson I, Vater CA, Stevens TH (1992). Morphological classification of the yeast vacuolar protein sorting mutants: evidence for a prevacuolar compartment in class E vps mutants.. Mol Biol Cell.

[36] Bowers K, Stevens TH (2005). Protein transport from the late Golgi to the vacuole in the yeast Saccharomyces cerevisiae.. Biochim Biophys Acta.

[37] Babst M, Katzmann DJ, Snyder WB, Wendland B, Emr SD (2002). Endosome-associated complex, ESCRT-II, recruits transport machinery for protein sorting at the multivesicular body.. Dev Cell.

[38] Babst M, Katzmann DJ, Estepa-Sabal EJ, Meerloo T, Emr SD (2002). Escrt-III: an endosome-associated heterooligomeric protein complex required for MVB sorting.. Dev Cell.

[39] Katzmann DJ, Babst M, Emr SD (2001). Ubiquitin-dependent sorting into the multivesicular body pathway requires the function of a conserved endosomal protein sorting complex, ESCRT-I.. Cell.

[40] Ballabio A, Gieselmann V (2009). Lysosomal disorders: from storage to cellular damage.. Biochim Biophys Acta.

[41] Bellettato CM, Scarpa M (2010). Pathophysiology of neuropathic lysosomal storage disorders.. J Inherit Metab Dis.

[42] Vitner EB, Platt FM, Futerman AH (2010). Common and uncommon pathogenic cascades in lysosomal storage diseases.. J Biol Chem.

[43] Poupetova H, Ledvinova J, Berna L, Dvorakova L, Kozich V, Elleder M (2010). The birth prevalence of lysosomal storage disorders in the Czech Republic: comparison with data in different populations.. J Inherit Metab Dis.

[44] Parenti G, Andria G, Ballabio A (2015). Lysosomal storage diseases: from pathophysiology to therapy.. Annu Rev Med.

[45] Begley DJ, Pontikis CC, Scarpa M (2008). Lysosomal storage diseases and the blood-brain barrier.. Curr Pharm Des.

[46] Barranger JM, Novelli EA (2001). Gene therapy for lysosomal storage disorders.. Expert Opin Biol Ther.

[47] Barton NW, Brady RO, Dambrosia JM, Di Bisceglie AM, Doppelt SH, Hill SC, Mankin HJ, Murray GJ, Parker RI, Argoff CE, Grewal RP, Yu K-T (1991). Replacement therapy for inherited enzyme deficiency - macrophage-targeted glucocerebrosidase for Gaucher’s Disease.. N Engl J Med.

[48] Schiffmann R, Kopp JB, Iii HAA, Sabnis S, Moore DF, Weibel T, Balow JE, Brady RO (2001). Enzyme replacement therapy in Fabry Disease: A randomized controlled trial.. JAMA.

[49] Pastores GM, Torres PA, Zeng BJ (2013). Animal models for lysosomal storage disorders.. Biochemistry (Mosc).

[50] Hirsch EC (2007). Animal models in neurodegenerative diseases.. J Neural Transm Suppl.

[51] Hemsley KM, Hopwood JJ (2010). Lessons learnt from animal models: pathophysiology of neuropathic lysosomal storage disorders.. J Inherit Metab Dis.

[52] Borbon I, Campbell E, Ke W, Erickson RP (2012). The role of decreased levels of Niemann-Pick C1 intracellular cholesterol transport on obesity is reversed in the C57BL/6J, metabolic syndrome mouse strain: a metabolic or an inflammatory effect?. J Appl Genetics.

[53] Jelinek D, Castillo JJ, Garver WS (2013). The C57BL/6J Niemann-Pick C1 mouse model with decreased gene dosage has impaired glucose tolerance independent of body weight.. Gene.

[54] Kovacs AD, Pearce DA (2015). Finding the most appropriate mouse model of juvenile CLN3 (Batten) disease for therapeutic studies: the importance of genetic background and gender.. Dis Models Mech.

[55] de Voer G, Peters D, Taschner PE (2008). Caenorhabditis elegans as a model for lysosomal storage disorders.. Biochim Biophys Acta.

[56] McCormick MA, Delaney JR, Tsuchiya M, Tsuchiyama S, Shemorry A, Sim S, Chou AC-Z, Ahmed U, Carr D, Murakami CJ, Schleit J, Sutphin GL, Wasko BM, Bennett CF, Wang AM, Olsen B, Beyer RP, Bammler TK, Prunkard D, Johnson SC, Pennypacker JK, An E, Anies A, Castanza AS, Choi E, Dang N, Enerio S, Fletcher M, Fox L, Goswami S, Higgins SA, Holmberg MA, Hu D, Hui J, Jelic M, Jeong K-S, Johnston E, Kerr EO, Kim J, Kim D, Kirkland K, Klum S, Kotireddy S, Liao E, Lim M, Lin MS, Lo WC, Lockshon D, Miller HA, Moller RM, Muller B, Oakes J, Pak DN, Peng ZJ, Pham KM, Pollard TG, Pradeep P, Pruett D, Rai D, Robison B, Rodriguez AA, Ros B, Sage M, Singh MK, Smith ED, Snead K, Solanky A, Spector BL, Steffen KK, Tchao BN, Ting MK, Wende HV, Wang D, Welton KL, Westman EA, Brem RB, Liu X, Suh Y, Zhou Z, Kaeberlein M, Kennedy BK (2015). A comprehensive analysis of replicative lifespan in 4,698 single-gene deletion strains uncovers conserved mechanisms of aging.. Cell Metab.

[57] Khurana V, Lindquist S (2010). Modelling neurodegeneration in Saccharomyces cerevisiae: why cook with baker’s yeast?. Nat Rev Neurosci.

[58] Menezes R, Tenreiro S, Macedo D, Santos CN, Outeiro TF (2015). From the baker to the bedside: yeast models of Parkinson’s disease.. Microb Cell.

[59] Kohlwein SD (2010). Obese and anorexic yeasts: Experimental models to understand the metabolic syndrome and lipotoxicity.. Biochim Biophys Acta.

[60] Ruggles KV, Garbarino J, Liu Y, Moon J, Schneider K, Henneberry A, Billheimer J, Millar JS, Marchadier D, Valasek MA, Joblin-Mills A, Gulati S, Munkacsi AB, Repa JJ, Rader D, Sturley SL (2014). A functional, genome-wide evaluation of liposensitive yeast identifies the “ARE2 required for viability” (ARV1) gene product as a major component of eukaryotic fatty acid resistance.. J Biol Chem.

[61] Pereira C, Coutinho I, Soares J, Bessa C, Leão M, Saraiva L (2012). New insights into cancer-related proteins provided by the yeast model.. FEBS J.

[62] Guaragnella N, Palermo V, Galli A, Moro L, Mazzoni C, Giannattasio S (2014). The expanding role of yeast in cancer research and diagnosis: insights into the function of the oncosuppressors p53 and BRCA1/2.. FEMS Yeast Res.

[63] Rakheja D, Narayan SB, Bennett MJ (2008). The function of CLN3P, the Batten disease protein.. Mol Genet Metab.

[64] Kalatzis V, Cherqui S, Antignac C, Gasnier B (2001). Cystinosin, the protein defective in cystinosis, is a H(+)-driven lysosomal cystine transporter.. EMBO J.

[65] Elmonem MA, Veys KR, Soliman NA, van Dyck M, van den Heuvel LP, Levtchenko E (2016). Cystinosis: a review.. Orphanet J Rare Dis.

[66] Attard M, Jean G, Forestier L, Cherqui S, van’t Hoff W, Broyer M, Antignac C, Town M (1999). Severity of phenotype in cystinosis varies with mutations in the CTNS gene: predicted effect on the model of cystinosin.. Hum Mol Genet.

[67] Cherqui S, Sevin C, Hamard G, Kalatzis V, Sich M, Pequignot MO, Gogat K, Abitbol M, Broyer M, Gubler M-C, Antignac C (2002). Intralysosomal cystine accumulation in mice lacking cystinosin, the protein defective in cystinosis.. Mol Cell Biol.

[68] Schnaper HW, Cottel J, Merrill S, Marcusson E, Kissane JM, Shackelford GD, So SKS, Nelson RD, Cole BR, Smith ML, Schneider JA (1992). Early occurrence of end-stage renal disease in a patient with infantile nephropathic cystinosis.. J Pediatr.

[69] Long WS, Seashore MR, Siegel NJ, Bia MJ (1990). Idiopathic Fanconi syndrome with progressive renal failure: a case report and discussion.. Yale J Biol Med.

[70] Gahl WA, Bashan N, Tietze F, Bernardini I, Schulman JD (1982). Cystine transport is defective in isolated leukocyte lysosomes from patients with cystinosis.. Science.

[71] Gahl WA, Thoene JG, Schneider JA (2002). Cystinosis.. N Engl J Med.

[72] Anikster Y, Shotelersuk V, Gahl WA (1999). CTNS mutations in patients with cystinosis.. Hum Mut.

[73] Jezegou A, Llinares E, Anne C, Kieffer-Jaquinod S, O’Regan S, Aupetit J, Chabli A, Sagne C, Debacker C, Chadefaux-Vekemans B, Journet A, Andre B, Gasnier B (2012). Heptahelical protein PQLC2 is a lysosomal cationic amino acid exporter underlying the action of cysteamine in cystinosis therapy.. Proc Natl Acad Sci USA.

[74] Liu B, Du H, Rutkowski R, Gartner A, Wang X (2012). LAAT-1 is the lysosomal lysine/arginine transporter that maintains amino acid homeostasis.. Science.

[75] Van Stralen KJ, Emma F, Jager KJ, Verrina E, Schaefer F, Laube GF, Lewis MA, Levtchenko EN (2011). Improvement in the renal prognosis in nephropathic cystinosis.. Clin J Am Soc Nephrol.

[76] Kimonis VE, Troendle J, Rose SR, Yang ML, Markello TC, Gahl WA (1995). Effects of early cysteamine therapy on thyroid function and growth in nephropathic cystinosis.. J Clin Endocrinol Metab.

[77] Markello TC, Bernardini IM, Gahl WA (1993). Improved renal function in children with cystinosis treated with cysteamine.. N Engl J Med.

[78] Reznik VM, Adamson M, Adelman RD, Murphy JL, Gahl WA, Clark KF, Schneider JA (1991). Treatment of cystinosis with cysteamine from early infancy.. J Pediatr.

[79] Kalatzis V, Serratrice N, Hippert C, Payet O, Arndt C, Cazevieille C, Maurice T, Hamel C, Malecaze F, Antignac C, Müller A, Kremer EJ (2007). The ocular anomalies in a cystinosis animal model mimic disease pathogenesis.. Pediatr Res.

[80] Racusen LC, Wilson PD, Hartz PA, Fivush BA, Burrow CR (1995). Renal proximal tubular epithelium from patients with nephropathic cystinosis: immortalized cell lines as in vitro model systems.. Kidney Int.

[81] Andrzejewska Z, Nevo N, Thomas L, Chhuon C, Bailleux A, Chauvet V, Courtoy PJ, Chol M, Guerrera IC, Antignac C (2016). Cystinosin is a component of the vacuolar H+-ATPase-Ragulator-Rag complex controlling mammalian Target of Rapamycin complex 1 signaling.. J Am Soc Nephrol.

[82] Ivanova EA, Heuvel LP van den, Elmonem MA, Smedt HD, Missiaen L, Pastore A, Mekahli D, Bultynck G, Levtchenko EN (2016). Altered mTOR signalling in nephropathic cystinosis.. J Inherit Metab Dis.

[83] Hardwick KG, Pelham HR (1990). ERS1 a seven transmembrane domain protein from Saccharomyces cerevisiae.. Nucleic Acids Res.

[84] Gao X-D, Wang J, Keppler-Ross S, Dean N (2005). ERS1 encodes a functional homologue of the human lysosomal cystine transporter.. FEBS Journal.

[85] Simpkins JA, Rickel KE, Madeo M, Ahlers BA, Carlisle GB, Nelson HJ, Cardillo AL, Weber EA, Vitiello PF, Pearce DA, Vitiello SP (2016). Disruption of a cystine transporter downregulates expression of genes involved in sulfur regulation and cellular respiration.. Biol Open.

[86] Kaur J, Bachhawat AK (2007). Yct1p, a novel, high-affinity, cysteine-specific transporter from the yeast Saccharomyces cerevisiae.. Genetics.

[87] Mole SE, Williams RE, Goebel HH (2005). Correlations between genotype, ultrastructural morphology and clinical phenotype in the neuronal ceroid lipofuscinoses.. Neurogenetics.

[88] Kyttälä A, Lahtinen U, Braulke T, Hofmann SL (2006). Functional biology of the neuronal ceroid lipofuscinoses (NCL) proteins.. Biochim Biophys Acta.

[89] No authors listed (1995). Isolation of a novel gene underlying Batten disease, CLN3. The International Batten Disease Consortium.. Cell.

[90] Schulz A, Kohlschütter A, Mink J, Simonati A, Williams R (2013). NCL diseases - clinical perspectives.. Biochim Biophys Acta.

[91] Hall NA, Lake BD, Dewji NN, Patrick AD (1991). Lysosomal storage of subunit c of mitochondrial ATP synthase in Batten’s disease (ceroid-lipofuscinosis).. Biochem J.

[92] Narayan SB, Rakheja D, Tan L, Pastor JV, Bennett MJ (2006). CLN3P, the Batten’s disease protein, is a novel palmitoyl-protein Δ-9 desaturase.. Ann Neurol.

[93] Warrier V, Vieira M, Mole SE (2013). Genetic basis and phenotypic correlations of the neuronal ceroid lipofusinoses.. Biochim Biophys Acta.

[94] Chandrachud U, Walker MW, Simas AM, Heetveld S, Petcherski A, Klein M, Oh H, Wolf P, Zhao W-N, Norton S, Haggarty SJ, Lloyd-Evans E, Cotman SL (2015). Unbiased cell-based screening in a neuronal cell model of Batten disease highlights an interaction between Ca2+ homeostasis, autophagy, and CLN3 protein function.. J Biol Chem.

[95] Järvelä I, Sainio M, Rantamäki T, Olkkonen VM, Carpén O, Peltonen L, Jalanko A (1998). Biosynthesis and intracellular targeting of the CLN3 protein defective in Batten disease.. Hum Mol Genet.

[96] Kama R, Kanneganti V, Ungermann C, Gerst JE (2011). The yeast Batten disease orthologue Btn1 controls endosome-Golgi retrograde transport via SNARE assembly.. J Cell Biol.

[97] Dobzinski N, Chuartzman SG, Kama R, Schuldiner M, Gerst JE (2015). Starvation-dependent regulation of golgi quality control links the TOR signaling and vacuolar protein sorting pathways.. Cell Rep.

[98] Phillips SN, Muzaffar N, Codlin S, Korey CA, Taschner PEM, de Voer G, Mole SE, Pearce DA (2006). Characterizing pathogenic processes in Batten disease: Use of small eukaryotic model systems.. Biochim Biophys Acta.

[99] Weber K, Pearce DA (2013). Large animal models for Batten disease: a review.. J Child Neurol.

[100] Pearce DA, Sherman F (1997). BTN1, a yeast gene corresponding to the human gene responsible for Batten’s disease, is not essential for viability, mitochondrial function, or degradation of mitochondrial ATP synthase.. Yeast.

[101] Pearce DA, Sherman F (1998). A yeast model for the study of Batten disease.. Proc Natl Acad Sci USA.

[102] Kim Y, Ramirez-Montealegre D, Pearce DA (2003). A role in vacuolar arginine transport for yeast Btn1p and for human CLN3, the protein defective in Batten disease.. Proc Natl Acad Sc USA.

[103] Golabek AA, Kida E, Walus M, Kaczmarski W, Michalewski M, Wisniewski KE (2000). CLN3 protein regulates lysosomal pH and alters intracellular processing of Alzheimer’s Amyloid-β protein precursor and cathepsin D in human cells.. Mol Genet Metab.

[104] Ramirez-Montealegre D, Pearce DA (2005). Defective lysosomal arginine transport in juvenile Batten disease.. Hum Mol Genet.

[105] Osorio NS, Carvalho A, Almeida AJ, Padilla-Lopez S, Leao C, Laranjinha J, Ludovico P, Pearce DA, Rodrigues F (2007). Nitric oxide signaling is disrupted in the yeast model for Batten disease.. Mol Biol Cell.

[106] Vitiello SP The yeast model for Batten disease: Genetic and physical interactions (Ph.D.). University of Rochester, United States - New York. 2008;. http://search.proquest.com/docview/304509717/abstract/118D2D38481241FAPQ/1.

[107] Chattopadhyay S, Pearce DA (2002). Interaction with Btn2p is required for localization of Rsg1p: Btn2p-mediated changes in arginine uptake in Saccharomyces cerevisiae.. Eukaryot Cell.

[108] Vitiello SP, Wolfe DM, Pearce DA (2007). Absence of Btn1p in the yeast model for juvenile Batten disease may cause arginine to become toxic to yeast cells.. Hum Mol Genet.

[109] Chattopadhyay S, Muzaffar NE, Sherman F, Pearce DA (2000). The yeast model for Batten Disease: Mutations in btn1, btn2, and hsp30 alter pH homeostasis.. J Bacteriol.

[110] Padilla-Lopez S, Langager D, Chan CH, Pearce DA (2012). BTN1, the Saccharomyces cerevisiae homolog to the human Batten disease gene, is involved in phospholipid distribution.. Dis Model Mech.

[111] Lloyd-Evans E, Platt FM (2010). Lipids on trial: the search for the offending metabolite in Niemann-Pick type C disease.. Traffic.

[112] Berger AC, Hanson PK, Wylie Nichols J, Corbett AH (2005). A Yeast Model system for functional analysis of the Niemann-Pick Type C protein 1 homolog, Ncr1p: A yeast model for NP-C Disease.. Traffic.

[113] Vanier MT (2010). Niemann-Pick disease type C.. Orphanet Rare Dis.

[114] Liscum L, Sturley SL (2004). Intracellular trafficking of Niemann-Pick C proteins 1 and 2: obligate components of subcellular lipid transport.. Biochim Biophys Acta.

[115] Alavi A, Nafissi S, Shamshiri H, Nejad MM, Elahi E (2013). Identification of mutation in NPC2 by exome sequencing results in diagnosis of Niemann-Pick disease type C.. Mol Genet Metab.

[116] Garver WS, Jelinek D, Meaney FJ, Flynn J, Pettit KM, Shepherd G, Heidenreich RA, Vockley CMW, Castro G, Francis GA (2010). The national Niemann-Pick Type C1 disease database: correlation of lipid profiles, mutations, and biochemical phenotypes.. J Lipid Res.

[117] Ohgami N, Ko DC, Thomas M, Scott MP, Chang CCY, Chang T-Y (2004). Binding between the Niemann-Pick C1 protein and a photoactivatable cholesterol analog requires a functional sterol-sensing domain.. Proc Natl Acad Sci USA.

[118] Ko DC, Binkley J, Sidow A, Scott MP (2003). The integrity of a cholesterol-binding pocket in Niemann-Pick C2 protein is necessary to control lysosome cholesterol levels.. Proc Natl Acad Sci USA.

[119] Pentchev PG (2004). Niemann-Pick C research from mouse to gene.. Biochim Biophys Acta.

[120] Pfeffer SR (2016). Clues to NPC1-mediated cholesterol export from lysosomes.. Proc Natl Acad Sci USA.

[121] Li X, Saha P, Li J, Blobel G, Pfeffer SR (2016). Clues to the mechanism of cholesterol transfer from the structure of NPC1 middle lumenal domain bound to NPC2.. Proc Natl Acad Sci USA.

[122] Malathi K, Higaki K, Tinkelenberg AH, Balderes DA, Almanzar-Paramio D, Wilcox LJ, Erdeniz N, Redican F, Padamsee M, Liu Y, Khan S, Alcantara F, Carstea ED, Morris JA, Sturley SL (2004). Mutagenesis of the putative sterol-sensing domain of yeast Niemann Pick C-related protein reveals a primordial role in subcellular sphingolipid distribution.. J Cell Biol.

[123] Vilaça R, Silva E, Nadais A, Teixeira V, Matmati N, Gaifem J, Hannun YA, Sá Miranda MC, Costa V (2014). Sphingolipid signalling mediates mitochondrial dysfunctions and reduced chronological lifespan in the yeast model of Niemann-Pick type C1: Sphingolipid signalling in the yeast model of NPC.. Mol Microbiol.

[124] Beltroy EP, Richardson JA, Horton JD, Turley SD, Dietschy JM (2005). Cholesterol accumulation and liver cell death in mice with Niemann-Pick type C disease.. Hepatology.

[125] Liao G, Yao Y, Liu J, Yu Z, Cheung S, Xie A, Liang X, Bi X (2007). Cholesterol Accumulation Is Associated with Lysosomal Dysfunction and Autophagic Stress in Npc1-/- Mouse Brain.. Am J Pathol.

[126] Tsuji T, Fujimoto M, Tatematsu T, Cheng J, Orii M, Takatori S, Fujimoto T (2017). Niemann-Pick type C proteins promote microautophagy by expanding raft-like membrane domains in the yeast vacuole.. Elife.

[127] Pipalia NH, Cosner CC, Huang A, Chatterjee A, Bourbon P, Farley N, Helquist P, Wiest O, Maxfield FR (2011). Histone deacetylase inhibitor treatment dramatically reduces cholesterol accumulation in Niemann-Pick type C1 mutant human fibroblasts.. Proc Natl Acad Sci USA.

[128] Wehrmann ZT, Hulett TW, Huegel KL, Vaughan KT, Wiest O, Helquist P, Goodson H (2012). Quantitative comparison of the efficacy of various compounds in lowering intracellular cholesterol levels in Niemann-Pick Type C fibroblasts.. PLoS ONE.

[129] Pipalia NH, Subramanian K, Mao S, Ralph H, Hutt DM, Scott SM, Balch WE, Maxfield FR (2017). Histone deacetylase inhibitors correct the cholesterol storage defect in most NPC1 mutant cells.. J Lipid Res.

[130] Nixon RA (2004). Niemann-Pick Type C disease and Alzheimer’s disease.. Am J Pathol.

[131] Wichit S, Hamel R, Bernard E, Talignani L, Diop F, Ferraris P, Liegeois F, Ekchariyawat P, Luplertlop N, Surasombatpattana P, Thomas F, Merits A, Choumet V, Roques P, Yssel H, Briant L, Missé D (2017). Imipramine inhibits Chikungunya virus replication in human skin fibroblasts through interference with intracellular cholesterol trafficking.. Sci Rep.

[132] Davey RA, Shtanko O, Anantpadma M, Sakurai Y, Chandran K, Maury W (2017). Mechanisms of filovirus entry.. Curr Top Microbiol Immunol.

[133] Feldmann H, Geisbert TW (2011). Ebola haemorrhagic fever.. Lancet.

[134] Dube D, Brecher MB, Delos SE, Rose SC, Park EW, Schornberg KL, Kuhn JH, White JM (2009). The primed Ebolavirus glycoprotein (19-Kilodalton GP1,2): sequence and residues critical for host cell binding.. J Virol.

[135] Kuhn JH, Radoshitzky SR, Guth AC, Warfield KL, Li W, Vincent MJ, Towner JS, Nichol ST, Bavari S, Choe H, Aman MJ, Farzan M (2006). Conserved receptor-binding domains of Lake Victoria marburgvirus and Zaire ebolavirus bind a common receptor.. J Biol Chem.

[136] Kaletsky RL, Simmons G, Bates P (2007). Proteolysis of the Ebola virus glycoproteins enhances virus binding and infectivity.. J Virol.

[137] Martinez O, Ndungo E, Tantral L, Miller EH, Leung LW, Chandran K, Basler CF (2013). A Mutation in the Ebola Virus Envelope Glycoprotein Restricts Viral Entry in a Host Species- and Cell-Type-Specific Manner.. J Virol.

[138] Carette JE, Raaben M, Wong AC, Herbert AS, Obernosterer G, Mulherkar N, Kuehne AI, Kranzusch PJ, Griffin AM, Ruthel G, Dal Cin P, Dye JM, Whelan SP, Chandran K, Brummelkamp TR (2011). Ebola virus entry requires the cholesterol transporter Niemann-Pick C1.. Nature.

[139] Cote M, Misasi J, Ren T, Bruchez A, Lee K, Filone CM, Hensley L, Li Q, Ory D, Chandran K, Cunningham J (2011). Small molecule inhibitors reveal Niemann-Pick C1 is essential for Ebola virus infection.. Nature.

[140] Wang H, Shi Y, Song J, Qi J, Lu G, Yan J, Gao GF (2016). Ebola Viral Glycoprotein bound to its endosomal receptor Niemann-Pick C1.. Cell.

[141] Gong X, Qian H, Zhou X, Wu J, Wan T, Cao P, Huang W, Zhao X, Wang X, Wang P, Shi Y, Gao GF, Zhou Q, Yan N (2016). Structural insights into the Niemann-Pick C1 (NPC1)-mediated cholesterol transfer and Ebola infection.. Cell.

[142] Naito T, Kiyasu Y, Sugiyama K, Kimura A, Nakano R, Matsukage A, Nagata K (2007). An influenza virus replicon system in yeast identified Tat-SF1 as a stimulatory host factor for viral RNA synthesis.. Proc Natl Acad Sci USA.

[143] Zheng Y-H, Plemenitas A, Fielding CJ, Peterlin BM (2003). Nef increases the synthesis of and transports cholesterol to lipid rafts and HIV-1 progeny virions.. Proc Natl Acad Sci USA.

[144] Barre-Sinoussi F, Chermann JC, Rey F, Nugeyre MT, Chamaret S, Gruest J, Dauguet C, Axler-Blin C, Vezinet-Brun F, Rouzioux C, Rozenbaum W, Montagnier L (1983). Isolation of a T-lymphotropic retrovirus from a patient at risk for acquired immune deficiency syndrome (AIDS).. Science.

[145] Popovic M, Sarngadharan MG, Read E, Gallo RC (1984). Detection, isolation, and continuous production of cytopathic retroviruses (HTLV-III) from patients with AIDS and pre-AIDS.. Science.

[146] Gallo RC, Salahuddin SZ, Popovic M, Shearer GM, Kaplan M, Haynes BF, Palker TJ, Redfield R, Oleske J, Safai B, Et A (1984). Frequent detection and isolation of cytopathic retroviruses (HTLV-III) from patients with AIDS and at risk for AIDS.. Science.

[147] Graham DRM, Chertova E, Hilburn JM, Arthur LO, Hildreth JEK (2003). Cholesterol depletion of Human Immunodeficiency Virus Type 1 and Simian Immunodeficiency Virus with β-Cyclodextrin inactivates and permeabilizes the virions: Evidence for virion-associated lipid rafts.. J Virol.

[148] Liao Z, Cimakasky LM, Hampton R, Nguyen DH, Hildreth JEK (2001). Lipid rafts and HIV pathogenesis: Host membrane cholesterol is required for infection by HIV Type 1.. AIDS Res Human Retroviruses.

[149] Liao Z, Graham DR, Hildreth JEK (2003). Lipid rafts and HIV pathogenesis: Virion-associated cholesterol is required for fusion and infection of susceptible cells.. AIDS Res Human Retroviruses.

[150] Tang Y, Leao IC, Coleman EM, Broughton RS, Hildreth JE (2009). Deficiency of niemann-pick type C-1 protein impairs release of human immunodeficiency virus type 1 and results in Gag accumulation in late endosomal/lysosomal compartments.. J Virol.

[151] Coleman EM, Walker TN, Hildreth JE (2012). Loss of Niemann Pick type C proteins 1 and 2 greatly enhances HIV infectivity and is associated with accumulation of HIV Gag and cholesterol in late endosomes/lysosomes.. Virol J.

[152] Norgan AP, Lee JRE, Oestreich AJ, Payne JA, Krueger EW, Katzmann DJ (2012). ESCRT-independent budding of HIV-1 Gag virus-like particles from Saccharomyces cerevisiae spheroplasts.. PLoS ONE.

[153] Andreola ML, Litvak S (2012). Yeast and the AIDS virus: the odd couple.. J Biomed Biotechnol.

[154] Meyre D, Delplanque J, Chèvre J-C, Lecoeur C, Lobbens S, Gallina S, Durand E, Vatin V, Degraeve F, Proença C, Gaget S, Körner A, Kovacs P, Kiess W, Tichet J, Marre M, Hartikainen A-L, Horber F, Potoczna N, Hercberg S, Levy-Marchal C, Pattou F, Heude B, Tauber M, McCarthy MI, Blakemore AIF, Montpetit A, Polychronakos C, Weill J, Coin LJM, Asher J, Elliott P, Järvelin M-R, Visvikis-Siest S, Balkau B, Sladek R, Balding D, Walley A, Dina C, Froguel P (2009). Genome-wide association study for early-onset and morbid adult obesity identifies three new risk loci in European populations.. Nat Genet.

[155] Kim SK, Park HJ, Lee JS, Park H-K, Jo DJ, Kim DH, Chung J-H, Kim M-J (2010). Association of Niemann-Pick disease, type C2 (NPC2) polymorphisms with obesity in Korean population.. Mol Cell Toxicol.

[156] Jelinek D, Castillo JJ, Richardson LM, Luo L, Heidenreich RA, Garver WS (2012). The Niemann-Pick C1 gene is downregulated in livers of C57BL/6J mice by dietary fatty acids, but not dietary cholesterol, through feedback inhibition of the SREBP pathway.. J Nutr.

[157] Jia L, Ma Y, Rong S, Betters JL, Xie P, Chung S, Wang N, Tang W, Yu L (2010). Niemann-Pick C1-Like 1 deletion in mice prevents high-fat diet-induced fatty liver by reducing lipogenesis.. J Lipid Res.

[158] Kurat CF, Natter K, Petschnigg J, Wolinski H, Scheuringer K, Scholz H, Zimmermann R, Leber R, Zechner R, Kohlwein SD (2006). Obese yeast: triglyceride lipolysis is functionally conserved from mammals to yeast.. J Biol Chem.

